# Effects of high temperature stress during anthesis and grain filling periods on photosynthesis, lipids and grain yield in wheat

**DOI:** 10.1186/s12870-020-02479-0

**Published:** 2020-06-09

**Authors:** M. Djanaguiraman, S. Narayanan, E. Erdayani, P. V. V. Prasad

**Affiliations:** 1 Department of Agronomy, Throckmorton Plant Science Center, Kansas State University, Manhattan, Kansas, 66506 USA; 2 Department of Crop Physiology, Tamil Nadu Agricultural University, Coimbatore, Tamil Nadu 641003 India; 3 Department of Plant and Environmental Sciences, 212 Biosystems Research Complex, Clemson University, Clemson, SC 29634 USA; 4 Department of Biotechnology, Indonesian Institute of Sciences, Cibinong Science Center, Kab. Bogor, 16911 Indonesia

**Keywords:** Grain number, High temperature, Individual grain weight, Lipids, Photosynthesis, Wheat

## Abstract

**Background:**

Short episodes of high temperature (HT) stress during reproductive stages of development cause significant yield losses in wheat (*Triticum aestivum* L.). Two independent experiments were conducted to quantify the effects of HT during anthesis and grain filling periods on photosynthesis, leaf lipidome, and yield traits in wheat. In experiment I, wheat genotype Seri82 was exposed to optimum temperature (OT; 22/14 °C; day/night) or HT (32/22 °C) for 14 d during anthesis stage. In experiment II, the plants were exposed to OT or HT for 14 d during the grain filling stage. During the HT stress, chlorophyll index, thylakoid membrane damage, stomatal conductance, photosynthetic rate and leaf lipid composition were measured. At maturity, grain yield and its components were quantified.

**Results:**

HT stress during anthesis or grain filling stage decreased photosynthetic rate (17 and 25%, respectively) and grain yield plant^− 1^ (29 and 44%, respectively), and increased thylakoid membrane damage (61 and 68%, respectively) compared to their respective control (OT). HT stress during anthesis or grain filling stage increased the molar percentage of less unsaturated lipid species [36:5- monogalactosyldiacylglycerol (MGDG) and digalactosyldiacylglycerol (DGDG)]. However, at grain filling stage, HT stress decreased the molar percentage of more unsaturated lipid species (36:6- MGDG and DGDG). There was a significant positive relationship between photosynthetic rate and grain yield plant^− 1^, and a negative relationship between thylakoid membrane damage and photosynthetic rate.

**Conclusions:**

The study suggests that maintaining thylakoid membrane stability, and seed-set per cent and individual grain weight under HT stress can improve the photosynthetic rate and grain yield, respectively.

## Background

Wheat (*Triticum aestivum* L.) is one of the important staple food crops in the world. Research indicates that most of the wheat-growing regions of the world are experiencing episodes of above-optimum temperatures leading to a significant decrease in grain yield [1–4]. Besides, IPCC [5] forecasted that in the future, crops would face short episodes of extreme temperatures, which will aggravate the negative effects of temperatures on grain yield [3, 4]. Wheat is sensitive to high temperature (HT) during reproductive stages compared to vegetative stages [6]. The OT for wheat during reproductive stages is between 15 and 20 °C [7, 8]. However, in wheat-growing regions of the world, an increased frequency of high daytime temperatures (> 34 °C) is expected [3]. If the occurrence of HT coincides with sensitive stages of wheat, it will cause significant negative impacts on grain yield. In field crops, an increase in temperature during critical growth stages may cause a yield reduction between 2.5 and 10% [9]. In wheat, 1 °C rise in minimum or maximum temperatures during cropping season could decrease the global wheat production by ~ 5.6% [1]. In another study, Barkley et al. [2] have shown that 1 °C increase in projected temperature during reproductive stages could decrease grain yield by 21%. Asseng et al. [3] have shown that global wheat production will decrease by 6% for each 1 °C increase of current mean temperature and will become more variable over time and space. Therefore, it is important to breed HT tolerant genotypes to sustain wheat production.

Leaf photosynthesis is severely affected by HT stress impacting plant growth and development [10]. Within the chloroplast, the photosystem II present in thylakoid membranes are highly sensitive to HT, and damages to thylakoid membrane decreased photosynthetic electron transfer, adenosine triphosphate phosphate synthesis and alterations in photochemical reactions [10, 11]. In addition, HT increases the production of reactive oxygen species (ROS) including the superoxide radical (O_2_^−^), hydrogen peroxide (H_2_O_2_), and lipid peroxidation, resulting in increased membrane damage [11, 12]. High temperature stress also induces thylakoid membrane swelling and leakiness [11], leading to the physical separation of chlorophyll light-harvesting complex II from the photosystem II core complex [13]. Ristic et al. [14] found a strong negative relationship (*r*^2^ = 0.78) between chlorophyll content and thylakoid membrane damage in winter wheat. Lower photosynthetic rate under HT stress in wheat is an interplay among thylakoid membrane damage, membrane lipid composition and oxidative damage to cell organelles [11].

Changes in membrane lipid composition and unsaturation levels are proposed to be an important mechanism of thermotolerance in wheat. Changes in membrane lipid unsaturation levels are required to prevent the phase transition of membranes to non-bilayer phases and to maintain membrane function and stability [15]. Studies on wheat leaves indicated that HT stress during the anthesis significantly decreased the total amount of monogalactosyldiacylglycerol (MGDG), phosphatidylglycerol (PG), phosphatidylcholine (PC) and phosphatidic acid (PA) [11, 12]. Apart from this, HT stress decreased the levels of more unsaturated lipids and increased the levels of less unsaturated and saturated lipids in both the heat susceptible and tolerant genotypes [12]. HT stress increased oxidized species of PC, and phosphatidylethanolamine (PE) in susceptible genotype [12]. Simultaneous changes in multiple lipid species under HT stress may be associated with the increases in activities of desaturating, oxidizing, glycosylating and acylating enzymes [16]. Lipid analyses in pollen grains of wheat have shown that 34:3 and 36:6 species of extraplastidic phospholipids [PC, PE, phosphatidylinositol (PI), PA and phosphatidylserine (PS)] dominated the lipid composition under optimum and HT conditions [17]. The unsaturation levels of these lipids were decreased through the decreases in the levels of 18:3 and increases in the levels of 16:0, 18:0, 18:1, and 18:2 acyl chains under HT stress [17]. The effects of HT on leaf lipids were quantified during anthesis and not during the grain filling stage, and comparative impacts were not quantified. In the present study, we take advantage of an electrospray ionization-tandem mass spectrometry (ESI-MS/MS) approach to quantitatively profile a wide range of leaf lipid molecular species under HT stress during anthesis and grain filling stages.

In general, plant yield is a function of plant architecture, photosynthetic efficiency, reproductive success and partitioning of carbohydrates to grain, and each of these components are vulnerable to HT in different ways [18]. In wheat, HT during anthesis stage decreased floret fertility by affecting pollen and pistil morphology and functions [19, 20]. The pollen morphological abnormalities include collapsed and desiccated, deeply pitted, rough exine wall, and loss of columellae head. Similarly, the style, stigma and ovary are desiccated and flaccid with less number of pollen grains adhered on the stigma [20]. In wheat, HT impairs viability, leading to poor fertilization [21, 22]. Similarly, HT decreased reproductive success (seed set) in major cereals like rice (*Oryza sativa* L.) [23], sorghum [*Sorghum bicolor* (L.) Moench] [24], and pearl millet [*Pennisetum glaucum* (L.) R. Br.] [25]. In wheat, HT during grain filling stage has been shown to decrease the grain yield through individual grain weight [20, 26, 27], which is associated with leaf senescence, and decreased grain-filling duration [3, 28, 29].

The objectives of this study are to quantify the effects of HT during anthesis and grain filling periods on photosynthesis, leaf lipidome, and yield-associated traits in wheat. We hypothesize that the decrease in photosynthesis during anthesis and grain filling stages were associated with changes in lipids and thylakoid membrane damage, decreased seed numbers and seed size leading to lower grain yields.

## Results

### Effects of temperature regime on physiological and yield traits

The mean data on various physiological traits recorded on 0, 2, 4, 6, 8, and 12 after the start of temperature treatments from the experiment I (HT during anthesis stage) and II (HT during grain filling stage) and its repeat (*n* = 36) are presented to get the overall effects of temperature treatment. Similarly, the mean values of yield and its components recorded in experiment I and II and its respective repeat (*n* = 20) are presented.

#### Experiment I: HT during anthesis stage

**Fig. 1:**
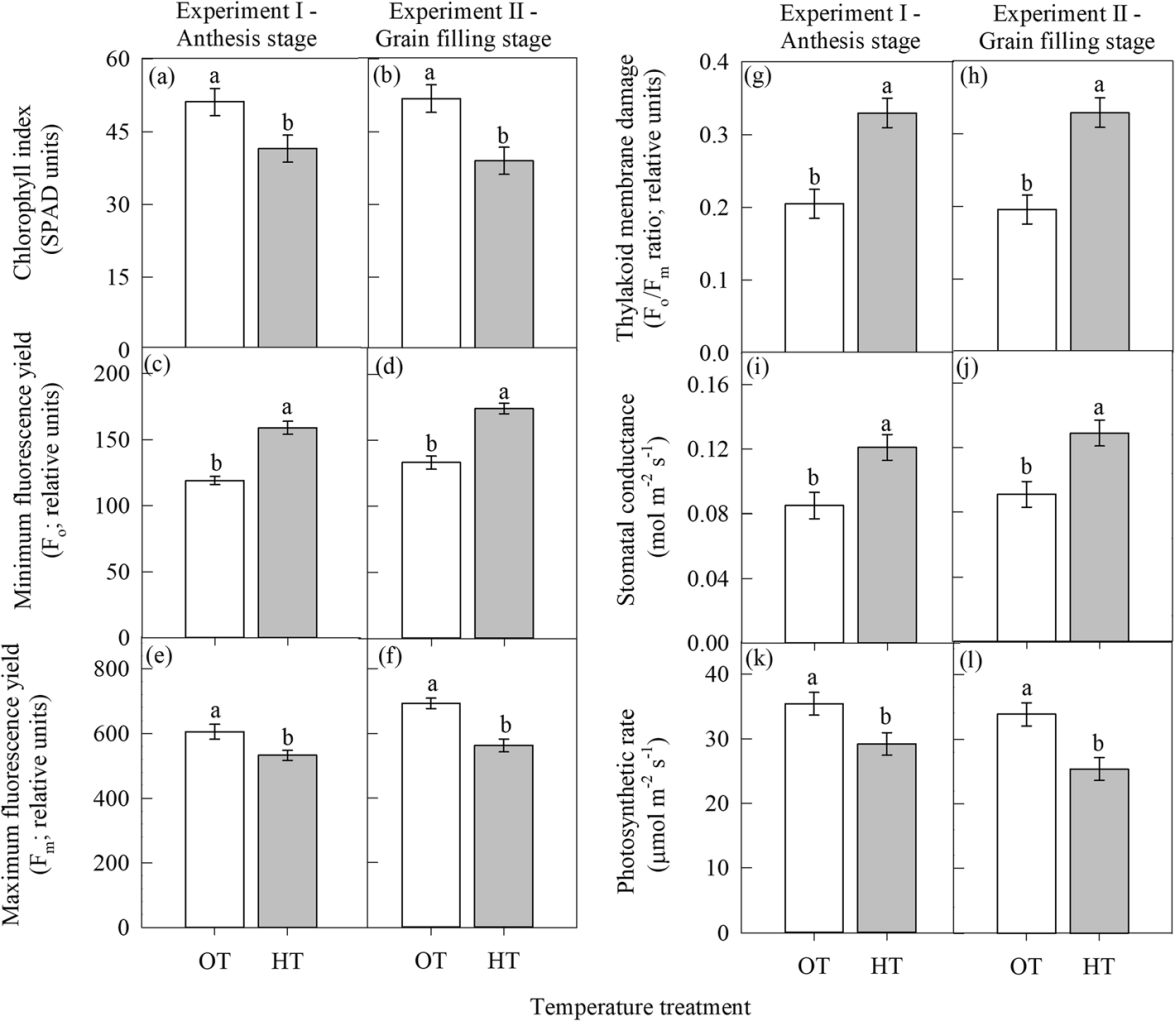
Effect of temperature regimes [optimal temperature (OT: 24/14 °C) and high temperature (HT: 32/22 °C)] on leaf physiological traits. **a** and **b** chlorophyll index (SPAD units), **c** and **d** minimum fluorescence yield (F_o_; relative units), **e** and **f** maximum fluorescence yield (F_m_; relative units), **g** and **h** thylakoid membrane damage (F_o_/F_m_ ratio; relative units), **i** and **j** stomatal conductance (mol m^− 2^ s^− 1^), and **k** and **l** photosynthetic rate (μmol m^− 2^ s^− 1^) during anthesis (experiment I) and grain filling (experiment II) stage, respectively. Values shown are LSMEAN ± standard error of LSMEAN [*n* = 36; 3 replications × 6 days of measurement (0, 2, 4, 6, 8, and 12 days after treatment imposition) x repeat of the experiment I and II (2)]. LSMEANS estimates with same letter are not significantly different at *P* ≤ 0.05

**Fig. 2:**
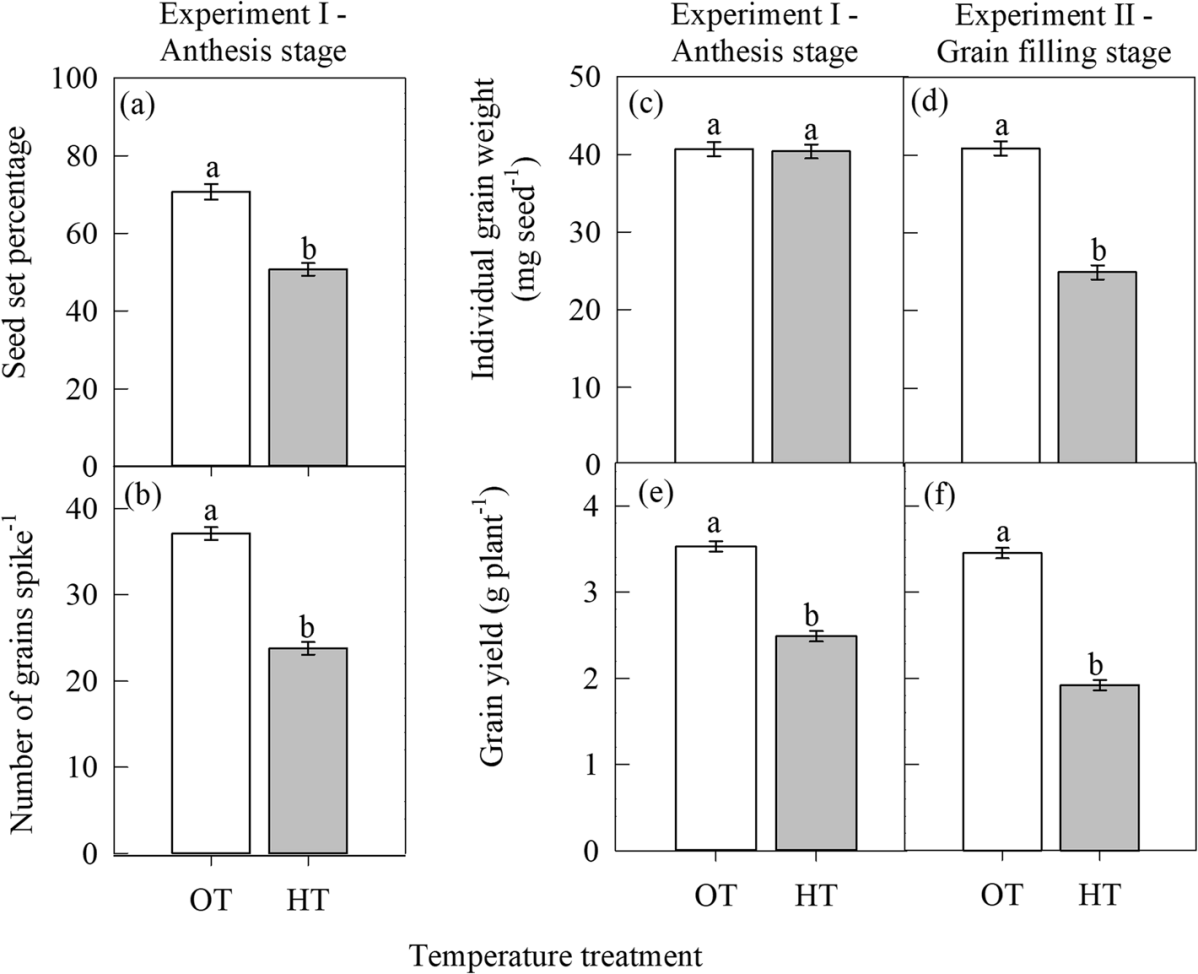
Effect of temperature regimes [optimal temperature (OT: 24/14 °C) and high temperature (HT: 32/22 °C)] on grain yield and its components. **a** seed set percentage, **b** number of grains spike^− 1^, **c** and **d** individual grain weight (mg seed^− 1^), and **e** and **f** grain yield (g plant^− 1^) during anthesis (experiment I) and grain filling (experiment II) stage, respectively. Values shown are LSMEAN ± standard error of LSMEAN (*n* = 20; 10 replications x repeat of the experiment I and II (2)). LSMEANS estimates with same letter are not significantly different at *P* ≤ 0.05

High temperature stress during anthesis stage (experiment I) significantly (*P* ≤ 0.05) decreased the chlorophyll index (SPAD units) by 19% compared to OT (Fig. 1a). Like the chlorophyll index, the maximum fluorescence yield (F_m_; relative units) and photosynthetic rate (μmol m^− 2^ s^− 1^) also decreased by 12 and 17%, respectively due to HT stress compared to OT (Fig. 1e and k). In contrast, HT during anthesis stage significantly (*P* ≤ 0.05) increased the minimum fluorescence yield (F_o_; relative units) by 34% (Fig. 1c), thylakoid membrane damage (F_o_/F_m_ ratio; relative units) by 61% (Fig. 1g), and stomatal conductance by 42% (Fig. 1i) than OT. High temperature during anthesis stage significantly (*P* ≤ 0.001) decreased seed set percentage by 28%, number of grains spike^− 1^ by 36%, and grain yield plant (g) by 29% compared to OT (Fig. 2a, b, and e).

#### Experiment II: HT during grain filling stage

Similar to experiment I, the HT stress during the grain filling stage (experiment II) significantly (*P* ≤ 0.05) decreased the chlorophyll index (25%), the maximum fluorescence yield (F_m_; relative units) (12%) and photosynthetic rate (25%) compared to OT (Fig. 1b, f, and l). However, HT significantly (*P* ≤ 0.05) increased the minimum fluorescence yield (F_o_; relative units), thylakoid membrane damage, and stomatal conductance by 31, 68, and 42%, respectively over OT (Fig. 1d, h and j). High temperature stress during grain filling stage significantly (*P* ≤ 0.001) decreased individual grain weight (mg seed^− 1^) by 39%, and plant grain yield by 44% over OT (Fig. 2d, and f).

### Effects of temperature regime on lipids composition

#### Experiment I: HT during anthesis stage

**Fig. 3:**
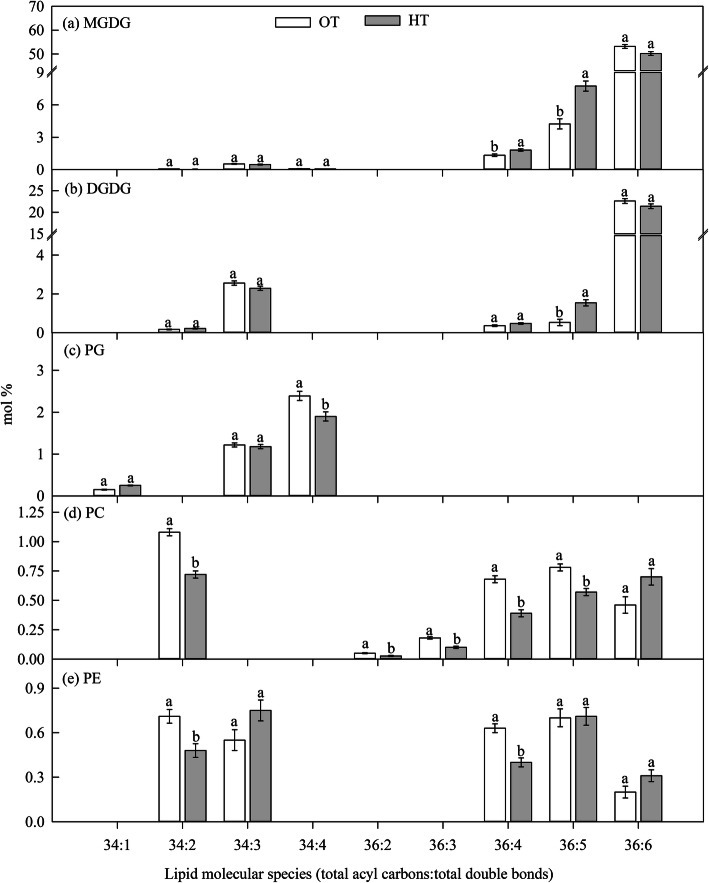
Effect of temperature regimes [optimal temperature (OT: 24/14 °C) and high temperature (HT: 32/22 °C)] during anthesis stage (experiment I) on lipid molecular species. Values shown are LSMEAN ± standard error of LSMEAN (*n* = 4). LSMEANS estimates with same letter within a lipid molecular species are not significantly different at *P* ≤ 0.05. MGDG, monogalactosyldiacylglycerol; DGDG, digalactosyldiacylglycerol; PG, phosphatidylglycerol; PC, phosphatidylcholine; and PE, phosphatidylethanolamine

Significant (*P* ≤ 0.05) effect of HT during anthesis stage (experiment I) was observed for the molar percentage of total PI (Table 1). High temperature stress increased the molar percentage of total PI by 23% over OT. Significant (*P* ≤ 0.05) increase in the molar percentage of less unsaturated lipid species containing two polyunsaturated acyl chains such as 36:5- (18:2/18:3 combination) MGDG and digalactosyldiacylgylcerol (DGDG) species was observed due to HT stress compared to OT (Fig. 3a, b). In contrary, the molar percentage of 34:2-, 36:2-, 36:3-, 36:4-, and 36:5- PC decreased significantly (*P* ≤ 0.05) under HT compared to OT (Fig. 3d). The proportion of more unsaturated lipid species, namely 36:6- (di18:3 combination) MGDG, DGDG, PC, and PE did not vary between OT and HT (Fig. 3a, b).

**Table 1:** Effect of temperature regimes [optimal temperature (24/14 °C) and high temperature (32/22 °C)] during anthesis (experiment I) and grain filling (experiment II) stages on proportion of various lipid classes. Values shown are LSMEAN ± standard error of LSMEAN (*n* = 4). The LSMEANS followed by same letter(s) within each growth stage are not statistically significant at *P* ≤ 0.05

Polar lipid	Growth stages			
	Experiment I: High temperature during anthesis stage		Experiment II: High temperature during grain filling stage	
	Optimum temperature	High temperature	Optimum temperature	High temperature
Total MGDG	59.64 ± 1.00^a^	60.76 ± 1.00^a^	61.88 ± 0.25^b^	64.60 ± 0.25^a^
Total DGDG	26.81 ± 0.58^a^	26.47 ± 0.58^a^	26.24 ± 0.33^a^	23.62 ± 0.33^b^
Total PG	4.68 ± 0.18^a^	4.20 ± 0.18^a^	5.17 ± 0.22^a^	5.12 ± 0.22^a^
Total PC	4.44 ± 0.21^a^	3.87 ± 0.21^a^	3.29 ± 0.09^a^	3.19 ± 0.09^a^
Total PE	2.98 ± 0.24^a^	2.81 ± 0.24^a^	2.07 ± 0.07^a^	1.96 ± 0.07^a^
Total PI	1.15 ± 0.08^b^	1.49 ± 0.08^a^	1.06 ± 0.06^a^	1.22 ± 0.06^a^
Total PS	0.10 ± 0.004^a^	0.12 ± 0.004^a^	0.07 ± 0.009^b^	0.11 ± 0.009^a^
Total PA	0.09 ± 0.05^a^	0.14 ± 0.05^a^	0.04 ± 0.007^a^	0.05 ± 0.007^a^

*MGDG* Monogalactosyldiacylglycerol, *DGDG* Digalactosyldiacylgylcerol, *PG* Phosphatidylglycerol, *PC* Phosphatidylcholine, *PE* Phosphatidylethanolamine, *PI* Phosphatidylinositol, *PS* Phosphatidylserine, and *PA* Phosphatidic acid

#### Experiment II: HT during grain filling stage

**Fig. 4:**
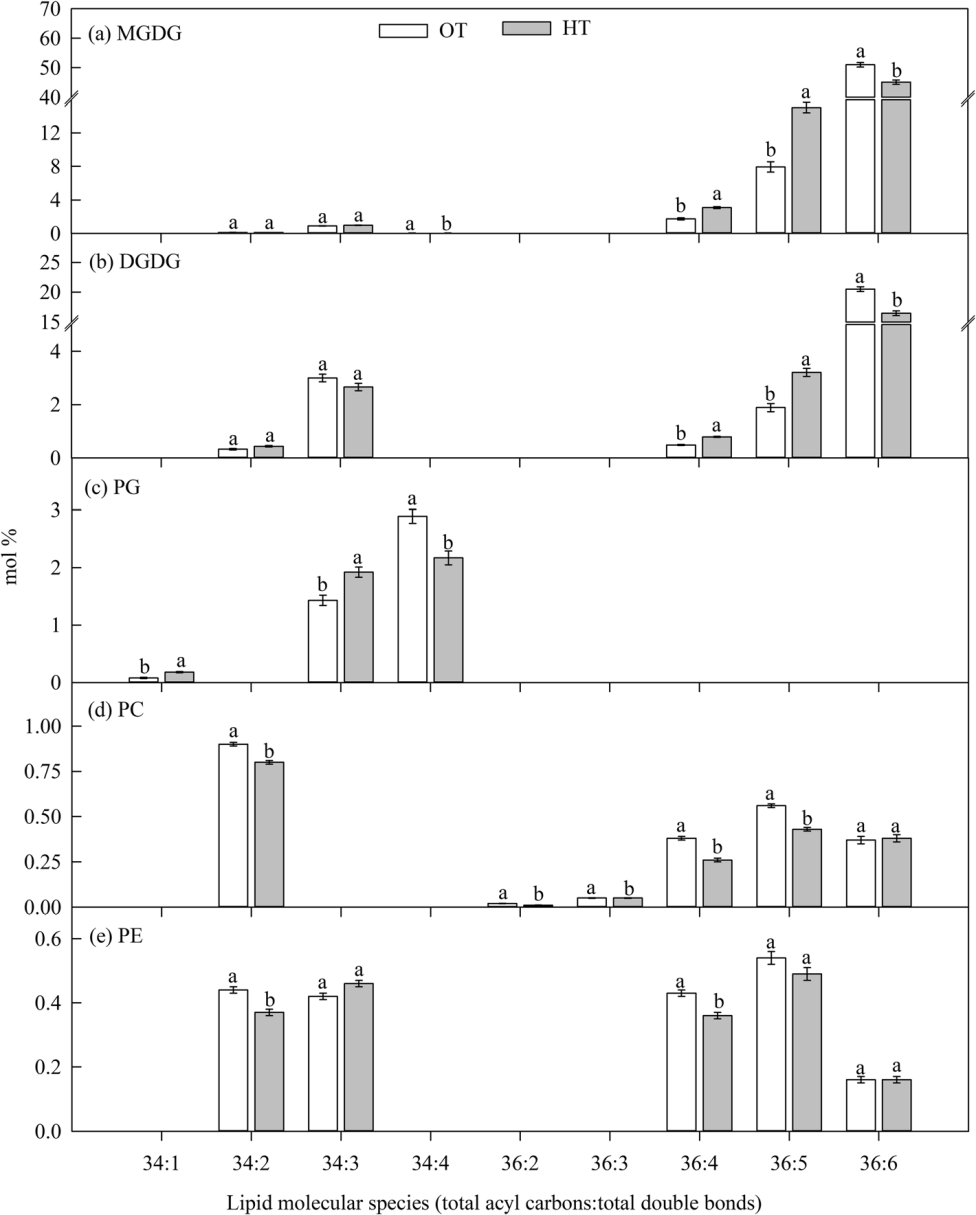
Effect of temperature regimes [optimal temperature (OT: 24/14 °C) and high temperature (HT: 32/22 °C)] during grain filling stage (experiment II) on lipid molecular species. Values shown are LSMEAN ± standard error of LSMEAN (*n* = 4). LSMEANS estimates with same letter within a lipid molecular species are not significantly different at *P* ≤ 0.05. MGDG, monogalactosyldiacylglycerol; DGDG, digalactosyldiacylglycerol; PG, phosphatidylglycerol; PC, phosphatidylcholine; and PE, phosphatidylethanolamine

Significant (*P* ≤ 0.05) effect of HT during the grain filling stage (experiment II) was observed for the molar percentage of total MGDG, DGDG, and PS (Table 1). High temperature stress increased the molar percentage of total- MGDG (4%) and PS (57%), and decreased the molar percentage of total DGDG (10%) compared to OT (Table 1). High temperature stress significantly (*P* ≤ 0.05) decreased the molar percentage of more unsaturated lipid species containing two polyunsaturated acyl chains such as 36:6- (di18:3 combination) MGDG and DGDG species over OT (Fig. 4a, b). In contrast, HT increased the molar percentage of less unsaturated lipid species containing two polyunsaturated acyl chains such as 36:5- (18:2/18:3 combination) MGDG and DGDG species or the amount of more saturated lipid species [containing one saturated acyl chain namely 34:1- (18:1/16:0 or 18:0/16:1 combination) PG species], and 36:4- (18:3/18:1 or 18:2/18:2 combination) species of MGDG and DGDG, and 34:3 PG (18:3/16:0 or 18:2/16:1 combination) over OT (Fig. 4a, b, c). However, the molar percentage of 34:4- (18:3/16:1) PG was significantly (*P* ≤ 0.05) decreased under HT than OT. All these variations indicate a decreased molar percentage of polyunsaturated acyl chain (18:3) or increased molar percentage of saturated acyl chain (16:0).

### Effects of temperature regime on lipid unsaturation level

#### Experiment I: HT during anthesis stage

Significant (*P* ≤ 0.05) effect of temperature regime during anthesis stage on the unsaturation index of plastidic and extraplastidic lipids was observed (Table 2). High temperature significant (*P* ≤ 0.05) decreased the unsaturation level of MGDG and PG over OT. However, the unsaturation level of PE was significant (*P* ≤ 0.05) increased under HT compared to OT (Table 2).

**Table 2:** Effect of temperature regimes [optimal temperature (24/14 °C) and high temperature (32/22 °C)] during anthesis (experiment I) and grain filling (experiment II) stages on unsaturation index of various lipid classes. Values shown are LSMEAN ± standard error of LSMEAN (*n* = 4). The LSMEANS followed by same letter(s) within each growth stage are not statistically significant at *P* ≤ 0.05

Polar lipid	Growth stages			
	Experiment I: High temperature during anthesis stage		Experiment II: High temperature during grain filling stage	
	Optimum temperature	High temperature	Optimum temperature	High temperature
MGDG	2.92 ± 0.006^a^	2.89 ± 0.006^b^	2.87 ± 0.007^a^	2.80 ± 0.007^b^
DGDG	2.79 ± 0.009^a^	2.77 ± 0.009^a^	2.72 ± 0.01^a^	2.66 ± 0.01^b^
PG	1.64 ± 0.008^a^	1.57 ± 0 .008^b^	1.69 ± 0.01^a^	1.59 ± 0.01^b^
PC	1.74 ± 0.02^a^	1.82 ± 0.02^a^	1.74 ± 0.01^a^	1.70 ± 0.01^a^
PE	1.81 ± 0.01^b^	1.89 ± 0.01^a^	1.87 ± 0.01^a^	1.86 ± 0.01^a^
PI	1.39 ± 0.01^a^	1.41 ± 0.01^a^	1.36 ± 0.004^a^	1.35 ± 0.004^a^
PS	1.29 ± 0.01^a^	1.33 ± 0.01^a^	1.31 ± 0.01^b^	1.36 ± 0.01^a^
PA	1.63 ± 0.02^a^	1.69 ± 0.02^a^	1.51 ± 0.03^a^	1.53 ± 0.03^a^

The unsaturation index of each lipid molecular species was calculated as the product of the amount of that lipid molecular species and the average number of double bonds per acyl chain, where the average number of double bonds per acyl chain was calculated by dividing the number of double bonds in the lipid molecular species by the number of acyl chains. Finally, the unsaturation index of a lipid head group class was calculated as the sum of the unsaturation indices of individual lipid molecular species in that class. *MGDG* Monogalactosyldiacylglycerol, *DGDG* Digalactosyldiacylgylcerol, *PG* Phosphatidylglycerol, *PC* Phosphatidylcholine, *PE* Phosphatidylethanolamine, *PI* Phosphatidylinositol, *PS* Phosphatidylserine, and *PA* Phosphatidic acid

#### Experiment II: HT during grain filling stage

Significant (*P* ≤ 0.05) effect of temperature regime during the grain filling stage on the unsaturation index of MGDG, DGDG, PG, and PS was observed (Table 2). The unsaturation level of MGDG, DGDG, and PG was decreased due to HT stress compared to OT. However, the unsaturation level of PS increased under HT compared to OT (Table 2).

### Relationship among photosynthetic rate, thylakoid membrane damage, grain yield and its components

**Fig. 5:**
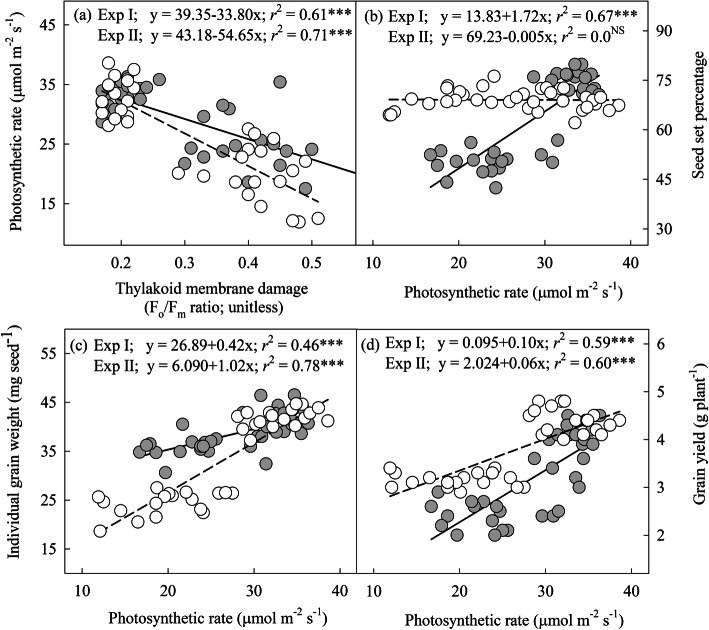
Relationship analysis. **a** photosynthetic rate (μmol m^− 2^ s^− 1^) as a function of thylakoid membrane damage (F_o_/F_m_ ratio; relative units), **b** seed set percentage as a function of photosynthetic rate (μmol m^− 2^ s^− 1^), **c** individual grain weight (mg seed^− 1^) as a function of photosynthetic rate (μmol m^− 2^ s^− 1^), and **d** grain yield (g plant^− 1^) as a function of photosynthetic rate (μmol m^− 2^ s^− 1^). Circle in gray and solid regression line indicates anthesis (experiment I) stage and circle in white and dotted regression line represent grain filling (experiment II) stage. *** indicates *P* ≤ 0.001

There was a negative linear relationship between thylakoid membrane damage and photosynthetic rate during anthesis (*r*^2^ = 0.61; *P* ≤ 0.001; Fig. 5a) and grain filling stage (*r*^2^ = 0.71; *P* ≤ 0.001; Fig. 5a). However, photosynthetic rate had a linear positive relationship with seed set percentage (*r*^2^ = 0.67; *P* ≤ 0.001; Fig. 5b), individual grain weight (*r*^2^ = 0.46; *P* ≤ 0.001; Fig. 5c), and grain yield (*r*^2^ = 0.59; *P* ≤ 0.001; Fig. 5d) during anthesis stage. Similarly, photosynthetic rate had a linear positive relationship with individual grain weight (*r*^2^ = 0.78; *P* ≤ 0.001; Fig. 5c), and grain yield (*r*^2^ = 0.60; *P* ≤ 0.001; Fig. 5d) during grain filling stage.

## Discussion

High temperature during anthesis or grain filling stage decreased the photosynthetic rate by decreasing thylakoid membrane integrity. The yield associated traits, namely seed set percentage and individual grain weight, were decreased due to HT during anthesis or grain filling stage, respectively. In addition, HT stress during anthesis or grain filling stage increased the molar percentage of less unsaturated lipid species (36:5- MGDG and DGDG). However, at the grain filling stage, HT stress decreased the molar percentage of more unsaturated lipid species (36:6- MGDG and DGDG). At both growth stages, there is a positive relationship among photosynthetic rate and grain yield plant^− 1^, and a negative association between thylakoid membrane damage and the photosynthetic rate.

High temperatures increased thylakoid membrane damage (Fig. 1g, h; F_o_/F_m_ ratio) because it is more sensitive to HT than other cell organelles [11]. An increased F_o_ value (1c, and d) under HT indicates damaged PS II reaction centres [30, 31], due to which the transfer of excitation energy from the antenna to the reaction centres will be lowered, resulting in an increased production of reactive oxygen species (ROS) [32, 33], and decreased production of NADPH_2_ [34, 35] which can potentially affect the carbon fixation process.

The chlorophyll molecule is primarily located on the thylakoid membranes as a complex with proteins of PS II and PS I, and damage to thylakoid membrane under HT may lead to chlorophyll loss [36, 37]. A strong negative relationship between thylakoid membrane damage and the photosynthetic rate at both anthesis and grain filling stage (Fig. 5a), indicates that the rate of thylakoid membrane damage under HT exceeds the rate of repair leading to net inhibition of photosynthetic rate [38]. An increase in growth temperature has decreased the photosynthetic rate during anthesis and grain filling stages (Fig. 1k, l); however, the former had less decrease over OT than later. This could be associated with leaf senescence phenomenon, which was activated during the grain filling stage in wheat [39].

Lipids such as MGDG tend to pack into a hexagonal phase or non-bilayer phases; in contrast, DGDG forms bilayers [15, 16, 40]. High temperature during grain filling stage decreased the molar percentage of total DGDG (Table 1), which might have resulted in a phase transition of membranes from the liquid crystalline phase to a hexagonal II or cubic phase leading to loss of membrane integrity. This indicates that during the grain filling stage, the membranes are highly prone to disintegration than anthesis stage. The similar extent of thylakoid membrane damage at both growth stages and lower molar percentage of total DGDG at grain filling compared to anthesis stage indicates that at anthesis stage the rate of repair of thylakoid membranes may be higher compared to grain filling stage, since, there was no variation in total plastidic lipids between OT and HT at anthesis stage (Table 1).

Taken together, HT stress caused a mixed effect in terms of lipid changes. The major effect is a reduction of desaturase activity as evident from the low molar percentage of more unsaturated lipids and high molar percentage of less unsaturated lipids (Figs. 3 and 4), and this may be an adaptive mechanism in leaves under HT to maintain the membranes fluidity [41, 42]. In wheat, glycolipids (MGDG and DGDG) are the major lipids, and 36:6- MGDG and DGDG (di18:3) are the major lipid species. These lipids decreased under HT during the grain filling stage (Fig. 4), because these species are highly vulnerable to peroxidation by ROS, which are produced under HT [11]. The decrease in unsaturation level was mainly due to the decrease in the polyunsaturated fatty acid (18:3) and an increase in less unsaturated fatty acids (18:2 and 18:1) and saturated fatty acids (16:0 and 18:0) (Fig. 4). This is in accordance with the findings of Narayanan et al. [12] and Djanaguiraman et al. [11]. These changes could be associated with terminal leaf senescence process during grain filling stage [43, 44], and also temperature optima for grain filling (21.3 °C) and anthesis stages (23 °C) [6]. Leaf and pollen lipidome is unique to each other, but the changes in lipid species under HT stress are similar and associated with HT tolerance in wheat [17].

High temperature during reproductive stages in wheat is associated with reductions in grain yield [22]. In wheat, 8 to 6 d before anthesis and anthesis stages are identified to be the most sensitive stages to HT stress [20]. Aliqing et al. [45] have observed that compared to control (optimum temperature) the reduction in spike grain weight under HT stress was greater in later-flowering tillers than early flowering tillers because the later-flowering tillers have experienced HT during gametogenesis stage, whereas, the early flowering tillers have experienced HT during the flowering stage. In the present study, HT during anthesis stage have decreased grain yield by lowering seed set per cent and grain numbers (Fig. 2a, b). The main physiological process happening during anthesis include dehiscence of anthers, pollen perception by stigma, pollen germination, pollen tube growth in the style, fertilization and embryo formation. Studies have shown that decreased functionality and structural abnormalities of pollen and/or pistil are the probable reasons for decreased seed numbers under HT [6, 20, 21, 46–48]. The individual grain weight was not affected under HT during anthesis stage because plants did not experience HT during the grain filling stage. Studies have shown that the rate of photosynthesis may also affect pollen tube growth in wheat [49], implying that photosynthetic rate during anthesis is critical in maintaining reproductive success. This was validated in this study by a significant linear relationship between photosynthetic rate and seed set percentage (Fig. 5b). High temperature during grain filling stage decreased grain yield plant^− 1^ by affecting the individual grain weight (Fig. 2d, f). In wheat, grain filling (weight) is linked with current assimilates production through photosynthesis [50] and/or remobilization of stored assimilates from vegetative tissues to developing reproductive tissues (grain) [51]. The reduction in grain yield plant^− 1^ under HT during grain filling stage could be due to accelerated development [52], and/or leaf senescence-associated with decreased photosynthetic rate [53–55].

## Conclusions

Under HT stress, changes in membrane lipid unsaturation levels were observed in the flag leaves at both anthesis and grain filling stage. The decrease in grain yield under HT during anthesis and grain filling stage was associated with grain numbers and individual grain weight, respectively. A positive relationship between photosynthetic rate and grain yield plant^− 1^ indicates that during anthesis and grain filling stage, maintaining greater photosynthetic rate is important for achieving higher seed numbers and seed size, ultimately influencing grain yield. With the recent developments in genomic research, targeting key genes involved in the synthesis of highly unsaturated lipid species can improve HT stress tolerance in wheat. Comprehensive gene expression studies on genes involved in thylakoid or pollen intrinsic/membrane lipid biosynthesis, degradation and remodeling will help in understanding the mechanism of tolerance. Understanding relationship among lipid molecular species, photosynthetic rate, and grain yield under HT stress will accelerate the molecular and physiological breeding for enhancing stress tolerance.

## Methods

Two independent experiments using spring wheat genotype Seri82 (seeds were obtained from Wheat Genetics Resource Center at Kansas State University; original seed source was International Maize and Wheat Improvement Center, Mexico) were conducted at controlled environment facilities available at the Department of Agronomy, Kansas State University, Manhattan, Kansas, USA.

### Plant husbandry and growth conditions

Wheat genotype Seri82 was grown as explained by Djanaguiraman et al. [11] in 1.8 L pots filled with commercial Sun Grow Metro Mix 200 potting soil (Hummert International, Topeka, Kansas, USA) and 10 g of controlled-release fertilizer (Osmocote Plus, N:P_2_O_5_:K_2_O = 15:9:12; Scotts, Marysville, Ohio, USA) in each pot. Forty plants were grown in a large indoor growth chamber (Conviron Model PGW40, Winnipeg, Manitoba, Canada) maintained at 24/14 °C (daytime maximum/nighttime minimum temperature), 14 h photoperiod, and ~ 70% relative humidity. The day and nighttime temperature were each held for 8 h, with a 4 h transition period. Cool white fluorescent lamps were used to produce photosynthetically active radiation (~ 900 mol m^− 2^ s^− 1^; Philips Lighting Co., Somerset, New Jersey, USA). After 21 d of emergence, three plants were retained per pot. To avoid sucking pests a systemic insecticide [Marathon 1% granular, with a.i.: Imidacloprid, 1-((6-chloro-3-pyridinyl) methyl)-N-nitro-2-imidazolidinimine, Hummert International, Topeka, Kansas, USA] was applied (four g pot^− 1^) [11]. Pots were well-watered (up to 100% pot capacity) by keeping in trays containing water ~ 2 cm deep from sowing to physiological maturity. Miracle-Gro, a water-soluble fertilizer (N:P_2_O_5_:K_2_O = 24:8:16; Scotts Miracle-Gro Products, Inc., Marysville, Ohio, USA) was added to the irrigation water (according to the manufacturer’s instructions) once in every 7 d from jointing (Feekes growth stage 6.0) to physiological maturity (Feekes growth stage 11.4). The pots were randomly arranged within the growth chamber and moved randomly on alternate days to avoid positional effects. Air temperature and relative humidity were monitored at 20-min intervals from sowing to physiological maturity. At the boot stage (Feekes growth stage 10.0), the main stem of each plant was tagged for measuring physiological, lipid, and yield traits.

### Temperature treatment imposition

#### Experiment I: HT during anthesis stage

At the anthesis stage (Feekes growth stage 10.5.1), two temperature regimes [optimum temperature (OT, 24/14 °C) and HT (32/22 °C)] were established randomly in two growth chambers (Conviron Model PGR15, Winnipeg, Manitoba, Canada). Ten pots were moved to each growth chamber. The plants were maintained in their respective temperature regime for 14 d. After exposing the plants to either OT or HT for 14 d during anthesis stage, the pots were moved back to the original growth chamber maintained at 24/14 °C and remained until physiological maturity.

#### Experiment II: HT during grain filling stage

During grain filling period (Feekes growth stage 10.5.4; 14 d after anthesis stage), 10 pots were moved to the growth chambers maintained at OT (24/14 °C) or HT (32/22 °C) to impose temperature treatment for 14 d. After exposing the plants to either OT or HT, the pots were moved back to the original growth chamber maintained at 24/14 °C and remained until physiological maturity.

### Physiological, lipids and yield traits

Out of 10 pots in each temperature regime during anthesis or grain filling period, 4 pots were used for measuring chlorophyll index, thylakoid membrane damage, stomatal conductance, and photosynthetic rate, 2 pots were used for collecting leaf samples for lipid analyses, and the remaining 4 pots were used for measuring grain yield and its associated components.

#### Chlorophyll index, thylakoid membrane damage, and gas exchange measurements

Chlorophyll index, chlorophyll *a* fluorescence, and gas exchange measurements were made on the attached flag leaves of tagged plants between 10:00 and 14:00 h, at OT and HT on days 0, 2, 4, 6, 8 and 12 after the start of temperature treatments in experiment I (HT during anthesis stage) and II (HT during grain filling stage). Out of four pots, three pots were randomly selected and one plant in each pot was tagged and used at each day of observation for measuring physiological traits. Chlorophyll index was measured in the middle portion of tagged flag leaves using a chlorophyll meter (SPAD-502, Spectrum Technologies, Plainfield, IL, USA) as explained by Djanaguiraman et al. [11]. Chlorophyll *a* fluorescence parameters [minimum fluorescence yield (F_o_) and maximum fluorescence yield (F_m_)] were measured on 30-min dark-adapted tagged flag leaves by using a modulated fluorometer (OS-30p, Opti-Science Inc., Hudson, New Hamshire, USA). Thylakoid membrane damage was determined as the ratio of F_o_/F_m_ (relative units). Photosynthesis and stomatal conductance were measured using a LICOR 6400 portable photosynthesis system (LICOR, Lincoln, Nebraska, USA) as described by Djanaguiraman et al. [11].

#### Lipid extraction and lipid profiling in leaves

Lipid composition was measured from four tagged flag leaves in both experiments I and II. The tagged flag leaves were collected for lipid extraction on the 10^th^ day of temperature treatment from each temperature regime. The middle one-third portion of the leaf was cut and immediately chopped into pieces, transferred into a 50-mL glass tube with a Teflon-lined screw cap (Thermo Fisher Scientific, Inc., Waltham, Massachusetts, USA), containing 6 mL of isopropanol (75 °C) with 0.01% butylated hydroxytoluene. Lipid extraction was performed as described by Narayanan et al. [12]. An automated electrospray ionization-tandem mass spectrometry approach was used for lipid profiling. Lipid unsaturation index was calculated as described by Narayanan et al. [12].

#### Yield and yield components

The yield and yield components were quantified from ten tagged plants in experiment I and II. At physiological maturity, the tagged spike on the main tiller of each plant from OT and HT was used for calculating seed set percentage, number of grains spike^− 1^ and individual grain weight (mg seed^− 1^) as described by Prasad and Djanaguiraman [20]. Similarly, the tagged and remaining spikes were harvested, dried in an incubator at 40 °C until constant weight was achieved. The spikes were hand threshed, and the grains were weighed to determine grain yield (g plant^− 1^).

### Statistical analyses

Each experiment I (HT during anthesis stage) and II (HT during grain filling stage) had two treatments namely OT and HT. The experiments I and II was repeated again with the same treatments and growth conditions mentioned earlier. The physiological and yield traits were recorded in both experiments; however, the lipids profiling was carried out in the repeat. The data were analysed in SAS 9.4 (SAS Institute Inc., Cary, North Carolina, USA) by using PROC MIXED procedures. For physiological traits, treatments were treated as class variable, days of observation and experiments were treated as random variable to get the overall effects of temperature treatment. The Tukey-Kramer adjustment was used to separate the treatment means. However, for grain yield and its associated traits, treatments were treated as class variable and the experiments were treated as random variable. The treatments were considered as class variable for lipid analyses. Regression analyses among physiological traits and grain yield were carried out by using the data from first and second run using PROC REG procedure of SAS.

## Data Availability

All the data on the present study has been included in the tables and/or figures form in this manuscript; and the datasets used and/or analyzed in this study are available from the corresponding author on reasonable request.

## Abbreviations

DBI: Double bond (unsaturation) index
DGDG: Digalactosyldiacylgylcerol;
ESI-MS/MS: Electrospray ionization-tandem mass spectrometry
HT: High temperature
MGDG: Monogalactosyldiacylglycerol
OT: Optimum temperature
PA: Phosphatidic acid
PC: Phosphatidylcholine
PE: Phosphatidylethanolamine
PG: Phosphatidylglycerol
PI: Phosphatidylinositol
PS: Phosphatidylserine
PS: Photosystem.
